# Menstrual Effluent Derived Immune Cell Composition Is Distinct in Women Using Contraceptives: A Pilot Study

**DOI:** 10.1111/aji.70145

**Published:** 2025-08-22

**Authors:** Aysel Gurbanova, Anne Stoverink, Imke M. B. van Wandeloo, Annemiek Nap, Nicole M. de Roos, Marien I. de Jonge, Janneke S. Hoogstad ‐ van Evert, Renate G. van der Molen

**Affiliations:** ^1^ Department of Laboratory Medicine Laboratory of Medical Immunology Radboud University Medical Center Nijmegen the Netherlands; ^2^ Division of Human Nutrition and Health Wageningen University Wageningen the Netherlands; ^3^ Department of Obstetrics and Gynecology Radboud University Medical Center Nijmegen the Netherlands; ^4^ Department of Obstetrics and Gynecology Amphia Breda the Netherlands

**Keywords:** combined oral contraception, copper intra‐uterine device, endometrial immune cells, menstrual effluent, uterine immune system

## Abstract

**Problem:**

Menstrual effluent (ME) is widely used to study immune‐related reproductive disorders. However, women using contraceptives are often excluded from such studies due to limited knowledge of their impact on the endometrial immune cell composition. This gap in knowledge restricts our ability to include women using contraceptives in the scientific investigations and account for potential immunological variations associated with contraceptive use. Therefore, we aim to investigate whether contraceptive use is associated with differences in the uterine immune system.

**Method of Study:**

ME was collected from women using combined oral contraceptives (COC, *n* = 5), a copper intrauterine device (IUD, *n* = 3), or no contraceptives (*n* = 5). Immune cells were isolated from ME and analyzed using flow‐cytometry. Data were analyzed using an unpaired *t*‐test, followed by multiple testing correction using the false discovery rate (FDR) method.

**Results:**

Women using COCs exhibited a marked reduction in the frequency of the immunoregulatory CD56^bright^CD16^−^ endometrial Natural Killer (eNK) cells compared to women using no contraceptives (30.1% ± 5.99 and 66.0% ± 10.6; *p* = 0.001). Additionally, women using copper IUD showed elevated frequencies of proliferating Ki‐67^+^CD8^+^ T cells compared to COC users and controls (19.8% ± 4.3, 2.69% ± 1.23, and 3.77% ± 4.4).

**Conclusions:**

This pilot study highlights the importance of considering contraceptive use when studying immune‐related reproductive problems.

## 1 Introduction

Menstrual effluent (ME) is a non‐invasive source of endometrium‐derived immune cells which closely resembles the immune cell composition and phenotypic characteristics of endometrial biopsies [1, 2, 3]. Several studies have used ME‐derived immune cells as a proxy for endometrial immune cell profiling to investigate reproductive problems such as recurrent pregnancy loss and endometriosis [2, 4]. Notably, ME—like endometrial biopsies—contains a high proportion of CD56^bright^CD16^−^ endometrial Natural Killer (NK) cells that are important for mucosal integrity, endometrial angiogenesis, and endometrial shedding [5]. Until now, most studies using ME have focused exclusively on women not using hormonal contraceptives or an intrauterine device (IUD). This is mainly due to the limited knowledge on how contraceptive use may influence the composition of immune cells in the endometrium. However, contraceptive use—especially combined oral contraceptives (COC)—is widespread, particularly among women with endometriosis, for whom COC is often prescribed as first‐line treatment [6].

COCs include a combination of artificial progesterone and estrogen, which inhibit ovulation by preventing the production and secretion of follicle‐stimulating hormone and luteinizing hormone from the pituitary gland [7]. When COCs are paused during a hormone‐free interval, withdrawal bleeding occurs as a response to the sudden drop in hormone levels. Estrogen and progesterone are known to have immunomodulatory functions, playing a role in inflammation and immune response to infections [8, 9, 10]. Previously, it was already shown that endometrial immune cells are responsive to hormonal changes [11]. Additionally, endometrial transcriptional profiling of women using hormonal IUD was shown to differentially express genes related to inflammation and immune activation compared to women not using any contraceptives [12]. These findings suggest that ME collected during a withdrawal bleeding from women using contraceptives and ME collected from women using a copper IUD may differ in immune composition from ME collected during natural cycles.

Therefore, the aim of this pilot study was to investigate the composition of ME‐derived immune cells in healthy volunteers using COC, or copper IUD, compared to controls not using any contraceptives. This will help determine whether ME remains a reliable proxy for endometrial immune assessment in women using contraceptives and inform future studies investigating endometrial immune cells using ME.

## 2 Methods

### 2.1 Study Participants

In this study, women with a regular menstrual cycle, aged 18 until maximum reproductive age of 45 with no known fertility issues, immunological diseases, or infectious diseases, using COC, a copper IUD or no medical contraception were compared. Women were excluded when they met one or more of the following exclusion criteria: active smoker, diagnosed with a gynecological condition, use of other hormonal medication or use of immunomodulating drugs. Women included in the control group, not using contraceptives, were only included if they had not been using COC or IUD for the last 6 months. The participants did not report any heavy menstrual bleeding or menstrual pain. COC use was stopped for 7 days to induce withdrawal bleeding. For clarity and consistency, we refer to this withdrawal bleeding as ME throughout the manuscript. This study was approved by the medical research ethics committee (Committee on Research Involving Human Subjects) of the region of Arnhem‐Nijmegen (file number CMO: NL8065209122). Informed consent was obtained from study participants before inclusion in the study. The cohort information, including age, BMI, duration of contraceptive use, and number of previous pregnancies, can be found in Table 1.

**TABLE 1:** Cohort characteristics.

Donor characteristics	Control (*n* = 5)	Oral contraceptive (*n* = 5)	Copper IUD (*n* = 3)
Type of contraceptive	NA	0.15 mg levonorgestrel and 0.03 mg ethinylestradiol (4/5)	NA
		2.5 mg nomegestrolacetaat and 1.5 mg estradiol (1/5)	
Age as median [range]	24 [24–40]	24 [22–27]	24 [23–34]
BMI (kg/m^2^) as median [range]	24.7 [19.8–28.7]	23.9 [20.5–29.4]	22.0 [18.7–23.9]
Duration of contraceptive use (in months) as median [range]	NA	84 [60–120]	11 [4–29]
Previous pregnancies	0/5	0/5	1/3

### 2.2 Sample Collection

ME was collected from study participants over a period of 36 h, in three 12‐h intervals, by means of a menstrual cup (AllMatters ApS, Copenhagen, Denmark) [1, 3, 4]. Each 12 h sample was transferred to ME collection tubes containing RPMI 1640 medium supplemented with sodium pyruvate [1 mM], GlutaMAX [2 mM], penicillin [100 U/mL], streptomycin [100 µg/mL] (all Thermo Fisher Scientific, Waltham, MA, USA), 10% human pooled serum (HPS) (manufactured in‐house), and 0.3% sodium citrate (Merck, Darmstadt, Germany).

### 2.3 Isolation of ME‐Derived Mononuclear Cells

ME‐derived mononuclear cells (MMCs) were isolated from ME as previously described [3, 4]. The volume of ME and the total number of cells isolated from ME were recorded. In brief, ME was filtered (70 µm) to remove mucus, followed by granulocyte depletion using RosetteSep (StemCell technologies Inc, Vancouver, Canada) and density gradient centrifugation (Lymphoprep).

### 2.4 Flow Cytometry

Immunophenotyping of MMCs was conducted with the Navios EX (Beckman Coulter, Fullerton, CA, USA) flow cytometry system. A minimum of 500 000 MMCs were stained using fluorochrome‐conjugated monoclonal antibodies (moABs) for 30 min at 4°C in the dark. Immunophenotyping of cells was done for general immune cell lineage markers, cytokine expression and markers associated with NK cells, B cells, T cells (including regulatory T cells), and monocytes. An overview of used moABs can be found in Table S1. Fixation and permeabilization of cells was performed according to manufacturer's instructions (eBioscience, San Diego, CA, USA). For intracellular cytokine detection MMCs were stimulated with phorbol 12‐myristate 13‐acetate (PMA) [12.5 ng/mL], Ionomycin [500 ng/mL], and Brefeldin‐A [5 µg/mL] for 4 h at 37°C and subsequently intra‐cellularly stained for IFN‐γ. Different flow cytometry panels were used to measure NK cells, T cells, B cells, monocytes, and intracellular cytokines.

### 2.5 Data Analysis

Flow cytometry data were analyzed and compensated using *Kaluza* software (Beckman Coulter Inc., Brea, CA). Immune cell subsets were manually gated, and their relative frequencies were extracted for statistical analysis. Gating strategies for immune cell subsets are described in Figures S1 and S2. Data visualization and statistical testing were performed using the *ggplot2* package in RStudio. The percentages of NK, T, B cells, and monocytes were derived from CD45^+^ lymphocytes. Group comparisons were conducted using unpaired *t*‐tests, with multiple testing correction applied using the false discovery rate (FDR) method. Results were considered statistically significant at an adjusted *p* value (FDR) < 0.05. Data are presented as percentages ± standard deviation (SD).

## 3 Results

### 3.1 The Immune Cell Composition of ME Is Distinct in Women Using COC and IUD Compared to Women Who Do Not Use Contraception

**FIGURE 1:**
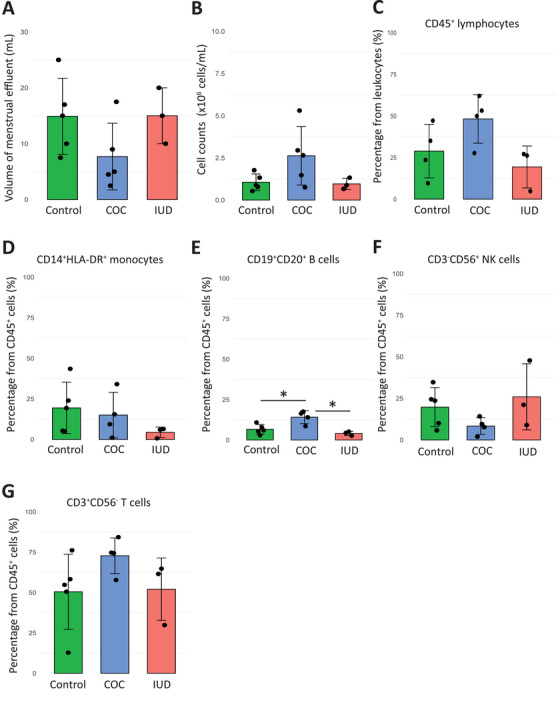
The immune cell composition of menstrual effluent in women using either combined oral contraceptive (COC) or a copper intra‐uterine device (IUD), and controls. (A) Bar plots indicating volume of ME in controls (*n* = 5, green), combined oral contraceptive (COC, *n* = 5, blue), and copper intrauterine device users (IUD, *n* = 3, red). (B) Bar plots indicating total cell counts isolated from ME indicated as ×10^6^ cells/mL of ME. (C) The bar plot represent the percentages of CD45^+^ lymphocytes in controls (green), COC (blue), and copper IUD (red) users. (D) Bar plots indicating frequencies of monocytes, (E) B cells, (F) NK cells, and (G) T cells. For statistical analysis, unpaired *t*‐test and FDR correction have been performed (*<0.05, **<0.005).

We first observed a trend toward a lower volume of ME collected in the COC group compared to the IUD and control groups. However, the total cell count per milliliter of ME was not reduced and even tended to be higher in the COC group (Figure 1A,B). Next, we examined the frequencies of CD45^+^ lymphocytes, which did not differ between the three groups (Figure 1C). The distribution of immune cell subsets per sample was also comparable across groups (Figure S3). A trend toward lower frequencies of CD14^+^HLA‐DR^+^ monocytes was noted in IUD users compared to controls (Figure 1D). The frequency of CD19^+^CD20^+^ B cells was significantly higher in the COC group compared to copper IUD users and controls (14.1% ± 4.0, 4.1% ± 1.3, and 6.6% ± 3.0; *p* = 0.032 and *p* = 0.035, respectively) (Figure 1E). Moreover, we observed a trend toward a reduced total CD56^+^CD3^−^ NK cell frequency in the COC group; however, this decrease was not significant (Figure 1F). We did not find any significant changes in CD56^−^CD3^+^ T cell frequencies (Figure 1G).

### 3.2 The Frequency of CD56^Bright^CD16^−^ Endometrial NK Cells Is Significantly Decreased in COC Users

**FIGURE 2:**
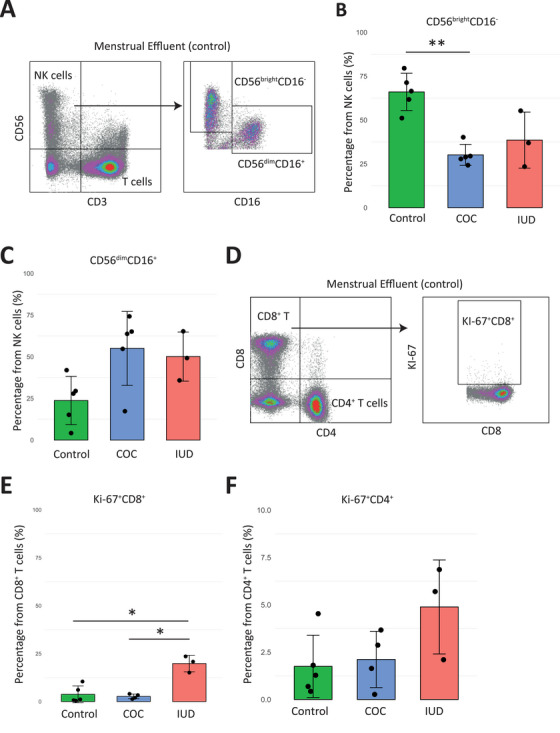
The frequency of endometrial NK cells of menstrual effluent decreases significantly in combined oral contraceptive (COC) users. (A) Dot plot representing gating strategy for NK cell subsets in ME of control. (B) Bar plot of endometrial NK cell percentages, also defined as CD56^bright^CD16^−^, and (C) CD56^dim^CD16^+^ NK cells in controls (*n* = 5, green), combined oral contraceptive (COC, *n* = 5, blue), and copper intrauterine device (IUD, *n* = 3, red). (D) Dot plot representing gating strategy for proliferating KI‐67^+^CD8^+^ T cells in ME of control. (E) Bar plots of CD8^+^ cytotoxic T cell and (F) CD4^+^ effector T cell percentages in each group. For statistical analysis, unpaired t‐test and FDR correction have been performed (*<0.05, **<0.005).

Next, we analyzed specific subsets of ME‐derived NK cells (Figure 2A). It is known that ME‐derived NK cells mainly contain CD56^bright^CD16^−^ endometrial NK (eNK) cells with immunoregulatory functions [5]. The analysis of the NK cell subsets showed significantly low abundance of the CD56^bright^CD16^−^ eNK cells in ME of COC users compared to controls (30.1% ± 5.99 and 66.0% ± 10.6; *p* = 0.001) (Figure 2B). In contrast, these women had higher frequencies of CD56^dim^CD16^+^ NK cells (Figure 2C). For IUD users a similar trend was observed. Additionally, CD56^bright^CD16^−^:CD56^dim^CD16^+^ ratios were significantly lower in COC and IUD users compared to controls (0.81 ± 0.34, 0.88 ± 0.28, and 5.60 ± 2.64, respectively).

### 3.3 The Frequency of KI‐67^+^CD8^+^ Cytotoxic T Cells Is Elevated in IUD Users

No significant differences in the frequency of endometrial B and T cell subsets among the three groups were observed (Figure S4). However, analysis of the proliferation status of endometrial T cells with proliferation marker Ki‐67 (Figure 2D) showed increased percentage of Ki‐67^+^ cytotoxic CD8^+^ T cells in IUD users compared to COC users and controls (19.8% ± 4.3, 2.69% ± 1.23, and 3.77% ± 4.4, respectively) (Figure 2E). We did not observe this increase for Ki‐67^+^ helper CD4^+^ T cells (Figure 2F).

## 4 Discussion

In this pilot study, we explored the association between contraceptive use and endometrial immune cell composition by analyzing ME from women using COCs, copper IUD, and those not using any contraception. We observed differences in immune cell subsets across all groups, including both hormonal and non‐hormonal contraceptive users. These preliminary findings suggest that contraceptive use and the method of contraception may differently influence the endometrial immune cell composition.

The human menstrual cycle is tightly regulated by ovarian sex hormones, primarily estrogen and progesterone [13]. During the proliferative phase, estradiol promotes the cellular proliferation of endometrial tissue [14]. In the subsequent secretory phase, progesterone induces the transformation of both endometrial stromal and immune cells into special decidual cells creating a receptive environment for embryo implantation and placentation [15]. In the absence of pregnancy, the withdrawal of estrogen and progesterone triggers menstruation [16]. This physiological process is altered in women using COCs due to the continuous presence of synthetic estrogen and progesterone. The bleeding is only induced upon cessation of COC, known as withdrawal bleeding. The impact of these synthetic hormones on the immune cells and immune cell infiltration during endometrial shedding remains poorly understood, making it challenging to interpret the ME from COC users as a reliable surrogate for endometrial immune cell composition.

Progesterone, plays a critical role in regulating immune cell infiltration in the uterus, among which the immunoregulatory CD56^bright^CD16^−^ eNK cells are the most abundant [11, 17]. eNK cells sequentially express surface markers representing recurrent cycle of differentiation in response to progesterone‐mediated release of stromal IL‐15 [11]. The frequencies of eNK cells fluctuate throughout the menstrual cycle, peaking during the late secretory phase and persisting throughout pregnancy [18]. Moreover, they are known to be involved in the establishment and maintenance of a successful pregnancy [19, 20]. In our study, we observed a significant reduction in the frequency of CD56^bright^CD16^−^ eNK cells in the ME of women using COCs. Bister et al. also showed significantly reduced frequencies of eNK cells expressing CD9 in the ME of COC users [21]. This reduction may be either due to the downstream effects of synthetic hormones on the endometrial tissue development or the altered hormone withdrawal pattern affecting the immune cell recruitment to the uterus during endometrial shedding.

Previously, analysis of endometrial transcriptomes of endometrial biopsies showed significant differences upon hormonal IUD use, while endometrial transcriptomes of women using copper IUD did not show significant differences [12]. We found an increased percentage of proliferating cytotoxic Ki‐67^+^CD8^+^ T cells in ME samples of copper IUD users, likely due to the influence of copper ions on the function and proliferation of immune cells [22]. The frequency of Ki‐67^+^CD8^+^ T cells was found to be higher in inflamed tissues [23]. Since this increase was not observed in COC users, our findings suggest that the copper IUD exerts a distinct and potentially more inflammatory effect on endometrial immune cells compared to COCs, indicating that hormonal and non‐hormonal contraceptive methods may differentially influence the endometrial immune cells.

The main limitation of our study is the small number of ME samples obtained across the three groups. Recruitment of COC users proved challenging, as participation required discontinuation of COC use for at least one week to allow withdrawal bleeding. Inclusion of IUD users was also limited due to the potential risk of IUD expulsion associated with ME collection [24]. Despite the small sample size, our data revealed clear group‐specific differences in immune cell composition. A second limitation was the low cell yield in ME samples, particularly from COC users, likely due to reduced bleeding volume. This limited the statistical power to detect differences in less abundant immune cell subsets. Additionally, a longitudinal approach—following women before and after initiating contraception—would have been more informative. However, this design proved difficult to implement, as participants using contraception were often reluctant to discontinue it, while those not using contraception were generally unwilling to start. Last, we did not assess the presence of infections in participants, which could have influenced the immune cell composition. Future studies should account for potential underlying infections when interpreting immune profiles in ME. Despite these limitations, our findings suggest that contraceptive use is associated with changes in endometrial immune cell composition and this influence varies depending on the type of contraception.

In conclusion, our study highlights the importance of considering contraceptive use when investigating ME for immune‐related reproductive health problems. ME may not be a reliable surrogate for assessing endometrial immune composition in contraceptive users. Further research is needed to better understand the underlying mechanisms of these contraceptive‐related immune changes and their potential implications for women's overall health and fertility.

## Conflicts of Interest

The authors declare that the research was conducted in the absence of any commercial or financial relationships that could be construed as a potential conflict of interest.

## Supporting information




**Supporting File**: aji70145‐sup‐0001‐SuppMat.docx

## Data Availability

The raw data supporting the conclusions of this article will be made available by the authors, without undue reservation.
