# Supplementary material for: Menstrual Effluent Derived Immune Cell Composition Is Distinct in Women Using Contraceptives: A Pilot Study
Source: Am J Reprod Immunol. 2025 Aug 22;94(2):e70145. doi: 10.1111/aji.70145 (PMC12372865; doi:10.1111/aji.70145)
Supplement: Supplementary file 1 — Supporting File: aji70145‐sup‐0001‐SuppMat.docx [file AJI-94-e70145-s001.docx]

**Supplementary Material**


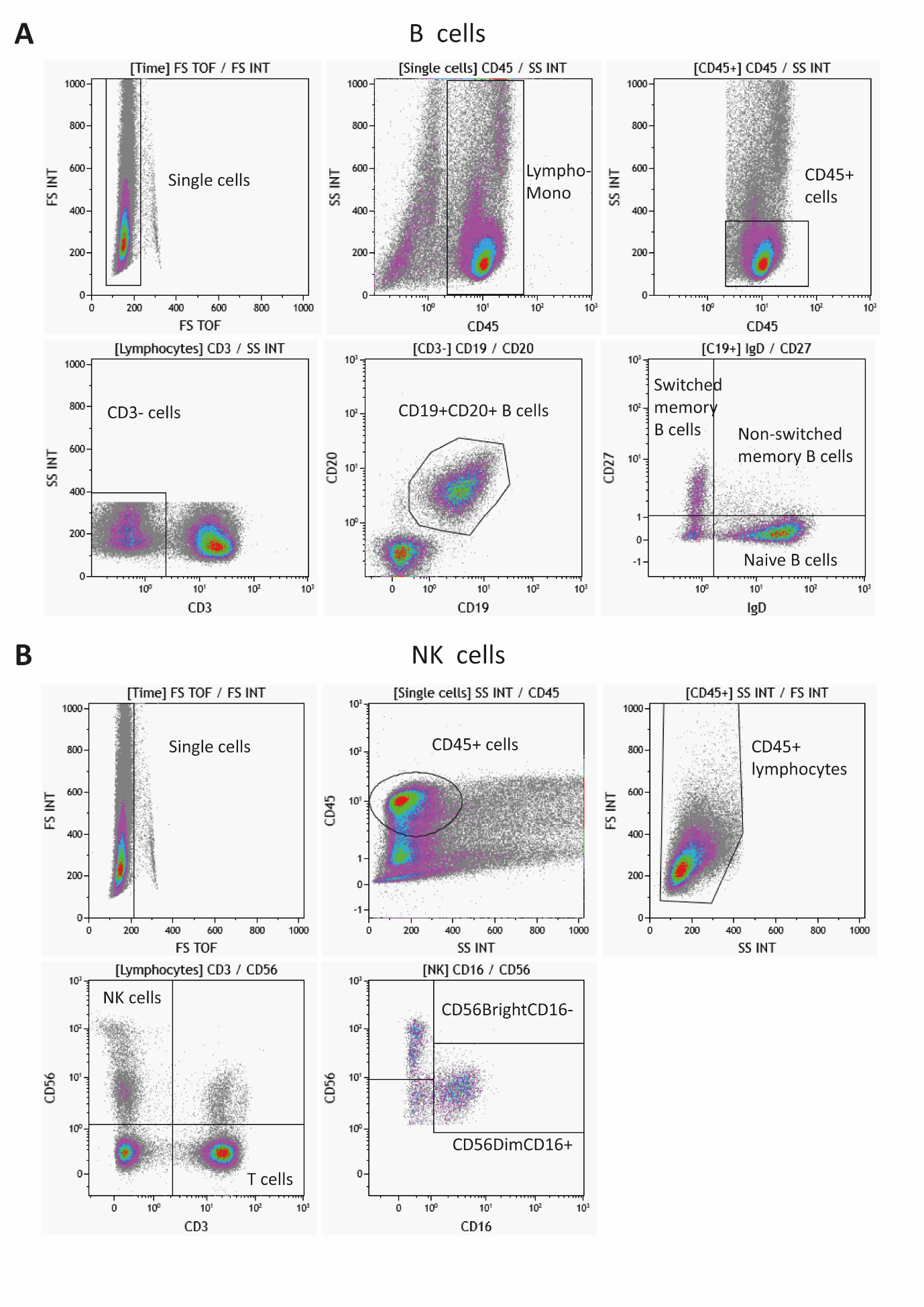
**Supplementary Figure 1. Gating strategies for B cells and NK cells in menstrual effluent.**


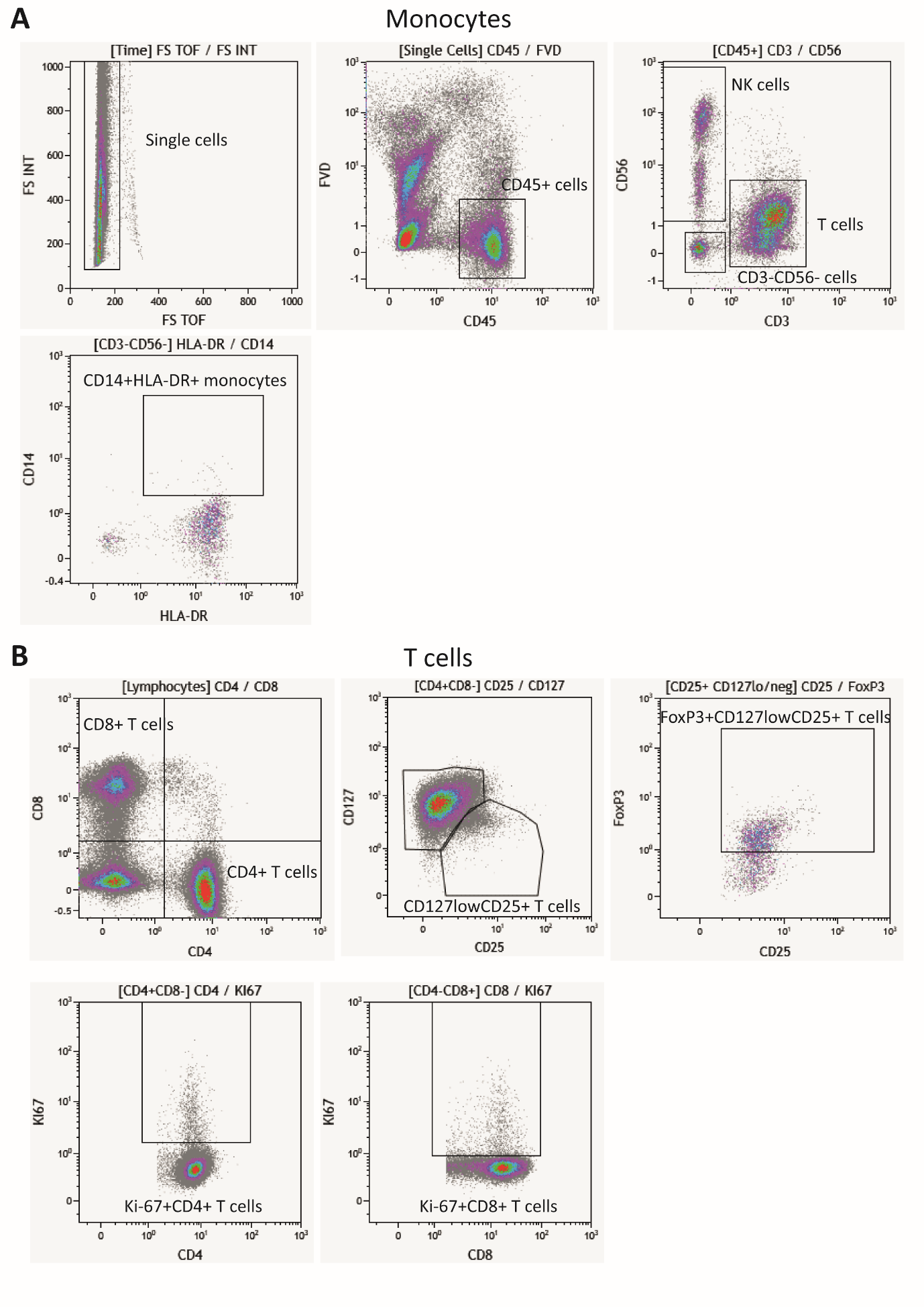


**Supplementary Figure 2. Gating strategies for Monocytes and T cells in menstrual effluent.**

**
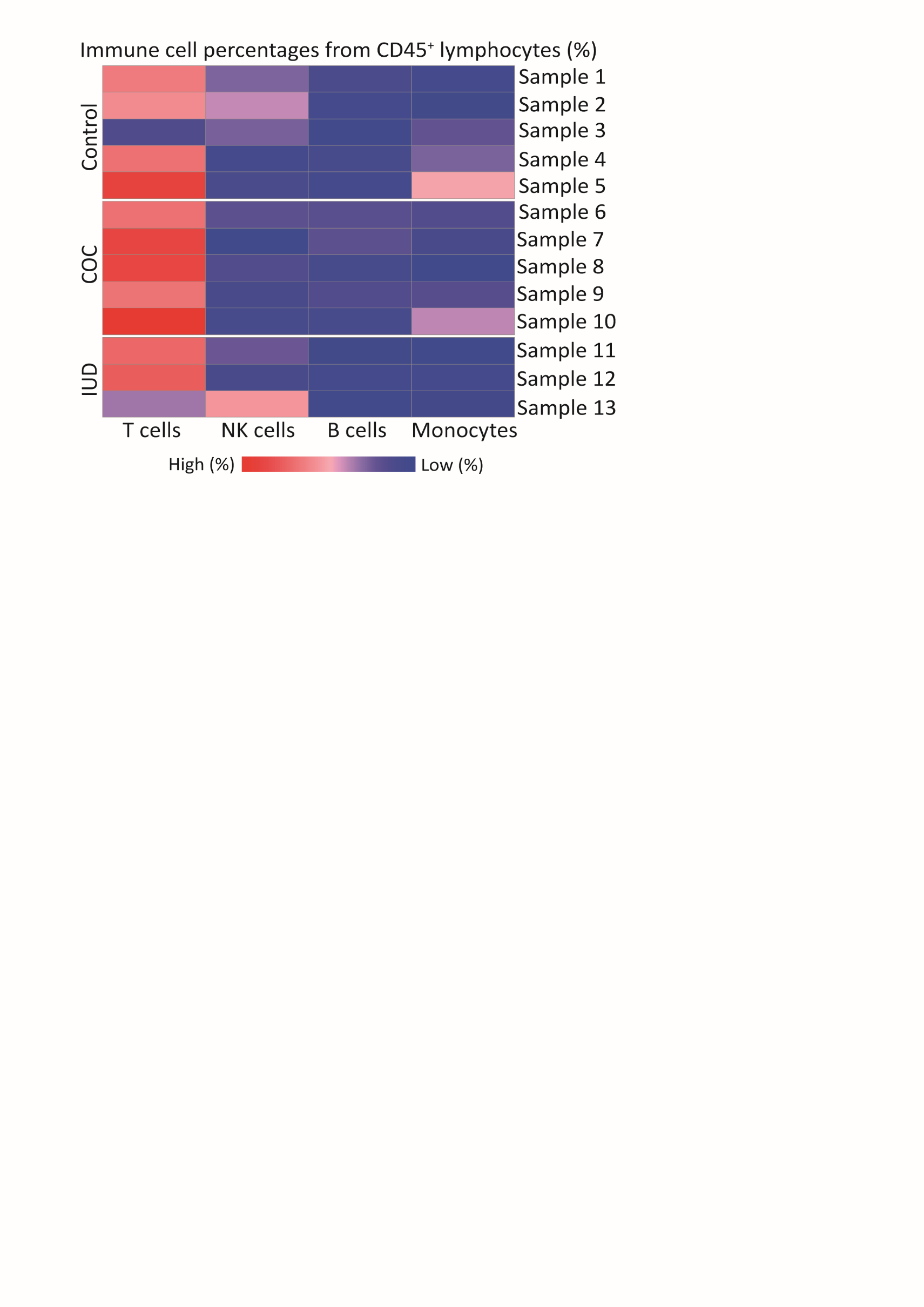
**

**Supplementary Figure 3. The immune cell composition of menstrual effluent in women using either combined oral contraceptive (COC) or intra-uterine device (IUD), and controls.** A) Heatmap representing percentages of immune cell subsets from CD45^+^ lymphocytes for each donor (controls, n=5; COC, n=5; copper IUD, n=3) (red-high percentage, blue-low percentage).

**
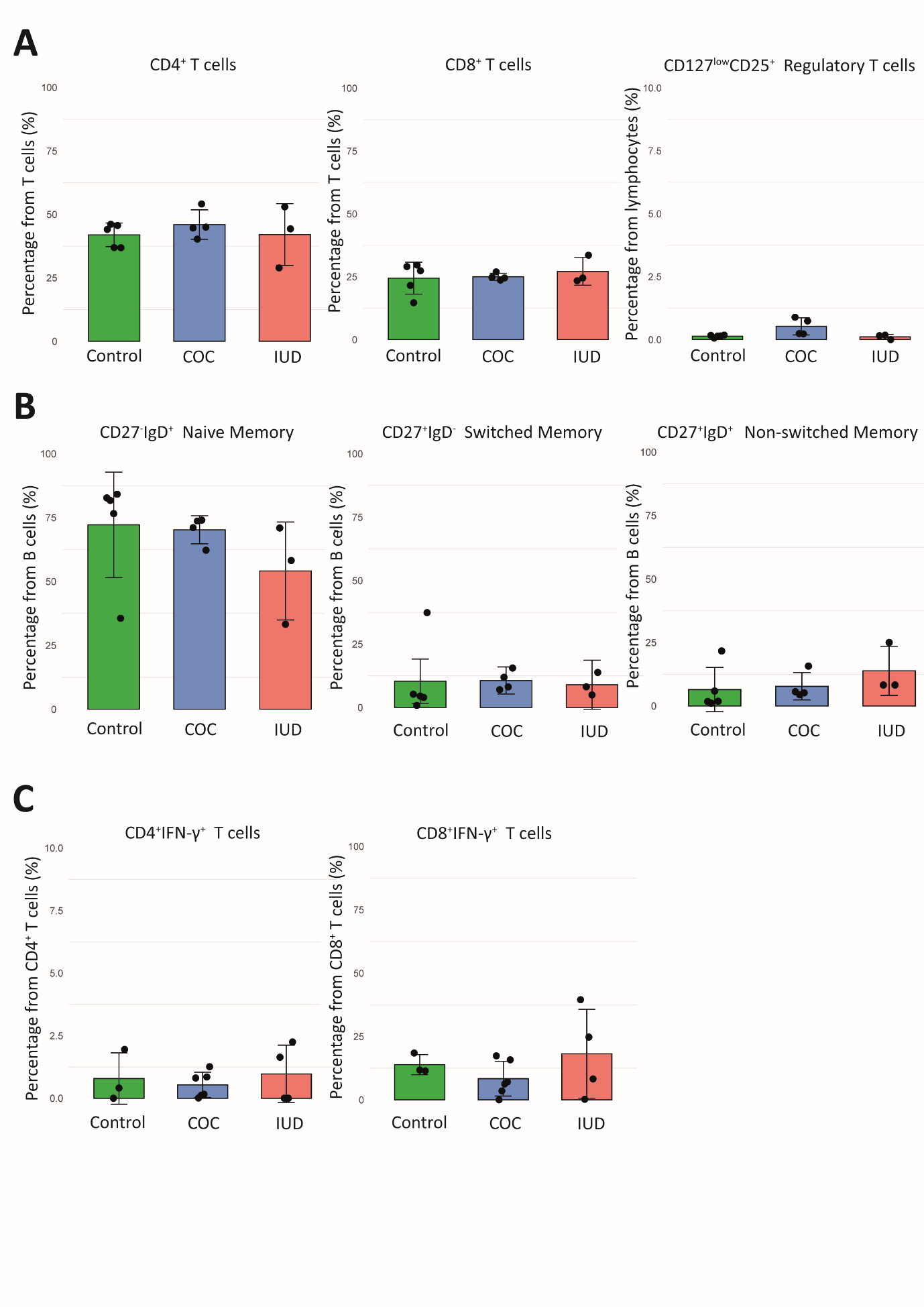
**

**Supplementary Figure 4. The immune cell composition of menstrual effluent in women using either combined oral contraceptive (COC) or intra-uterine device (IUD), and controls.** A) Bar plots indicating percentages of helper (CD3^+^CD8^-^CD4^+^), cytotoxic (CD3^+^CD8^+^CD4^-^) and regulatory (CD3^+^CD4^+^CD127^low^CD25^high^FoxP3^+^) T cells in controls (n=5, green), combined oral contraceptive (COC, n=5, blue), and copper intrauterine device (IUD, n=3, red). B) Bar plots indicating percentages of naïve (CD19^+^CD27^-^IgD^+^), switched-memory (CD19^+^CD27^+^IgD^-^) and non-switched memory (CD19^+^CD27^+^IgD^+^) B cells. C) Bar plots of IFN-γ producing Ki67^+^CD4^+^ helper T cell and Ki67^+^CD8^+^ cytotoxic T cell percentages in each group. For statistical analysis, unpaired t-test and FDR correction have been performed (*<0.05, **<0.005).

**Supplementary Table 1| List of monoclonal antibodies used for flow cytometry**
